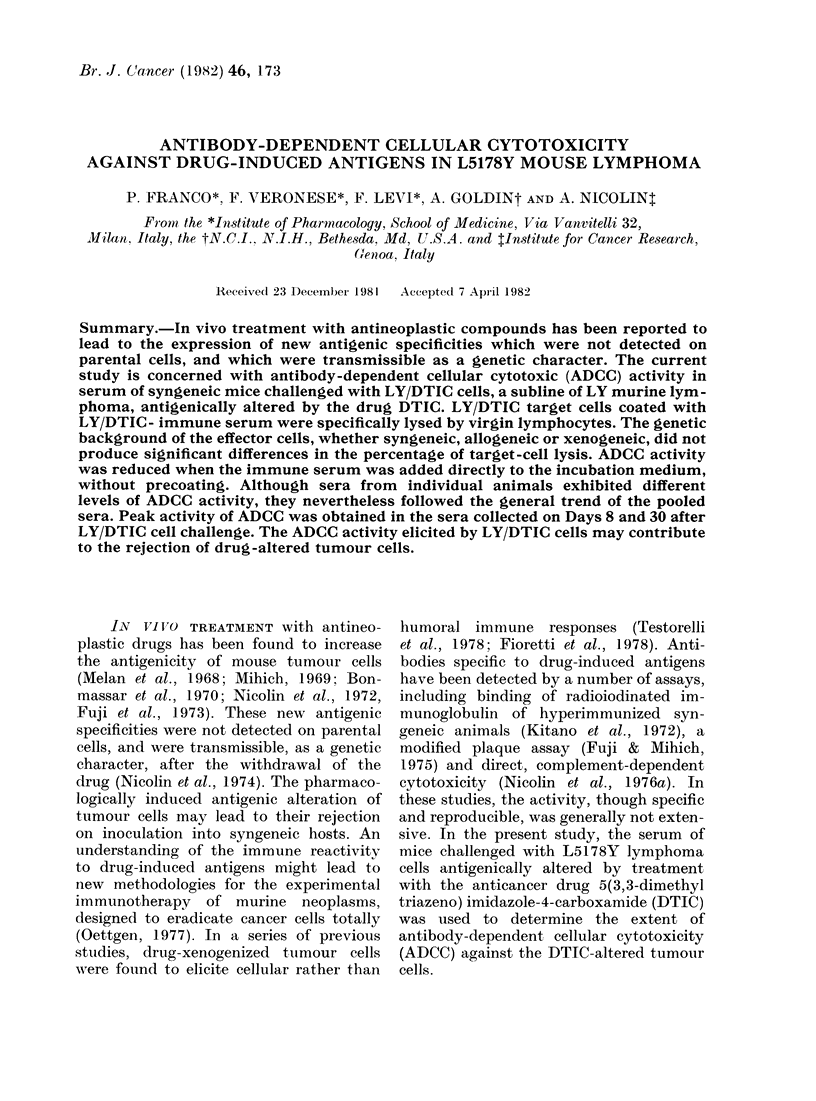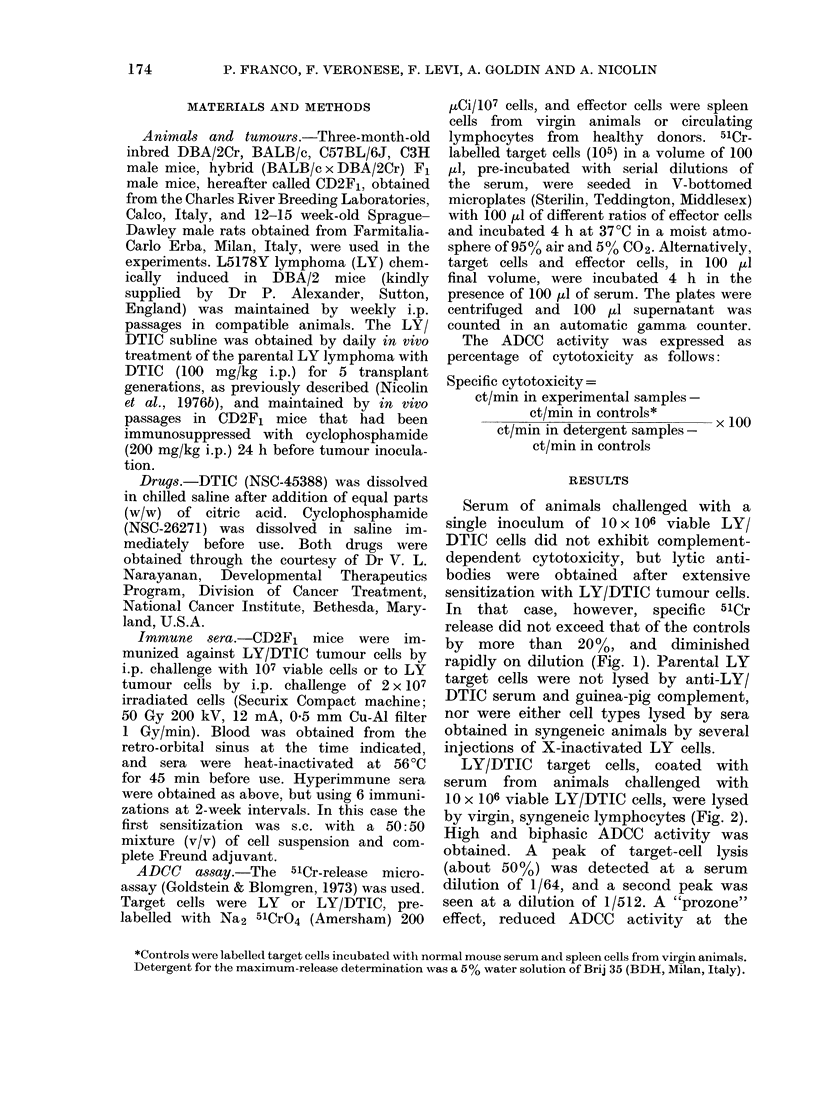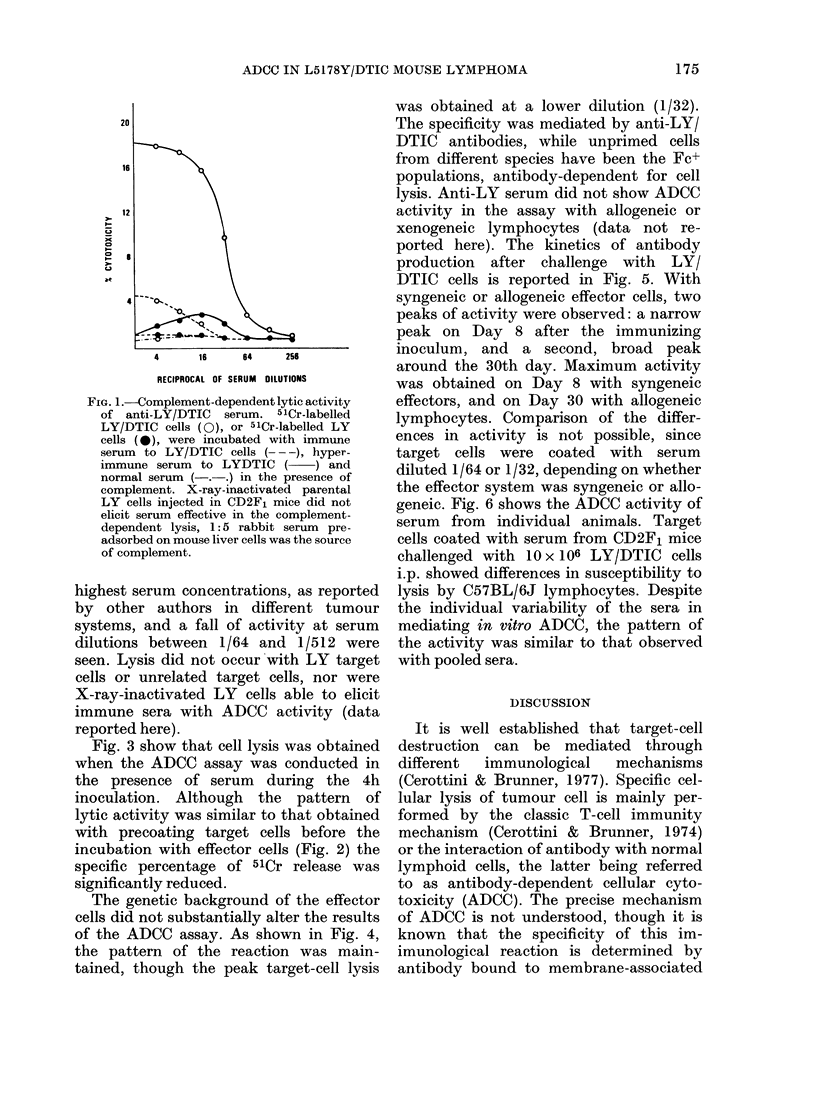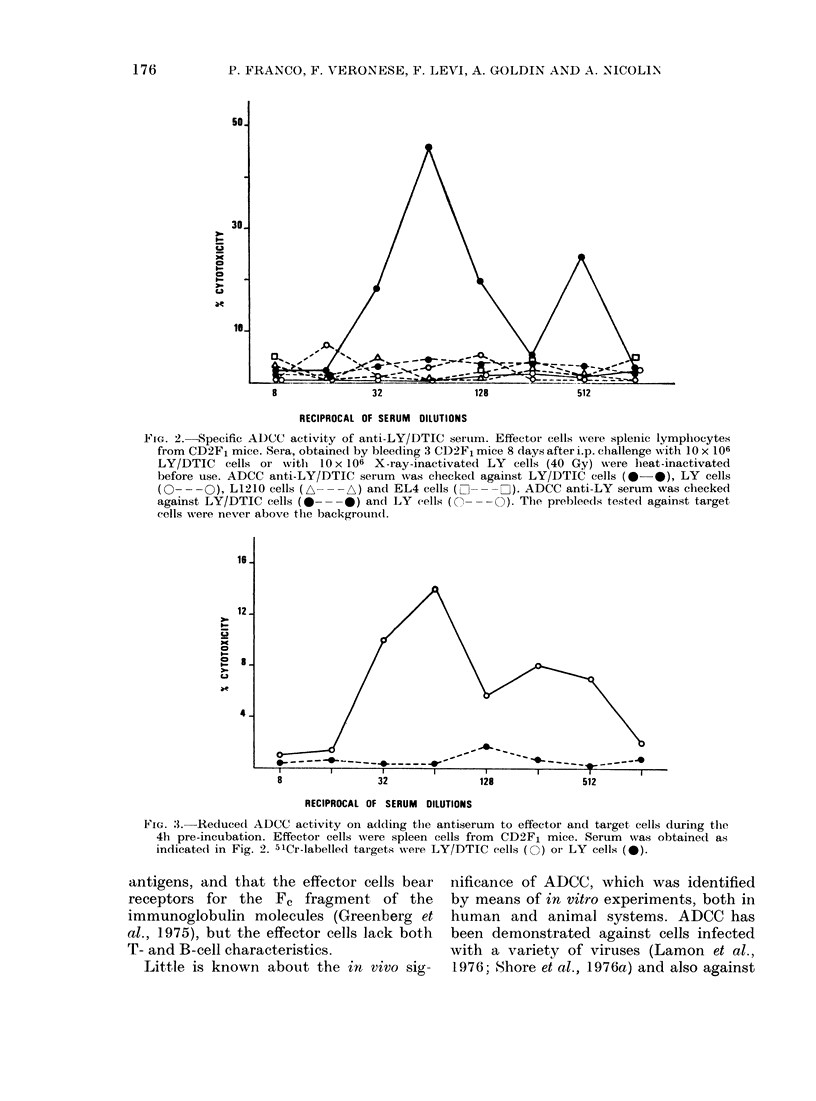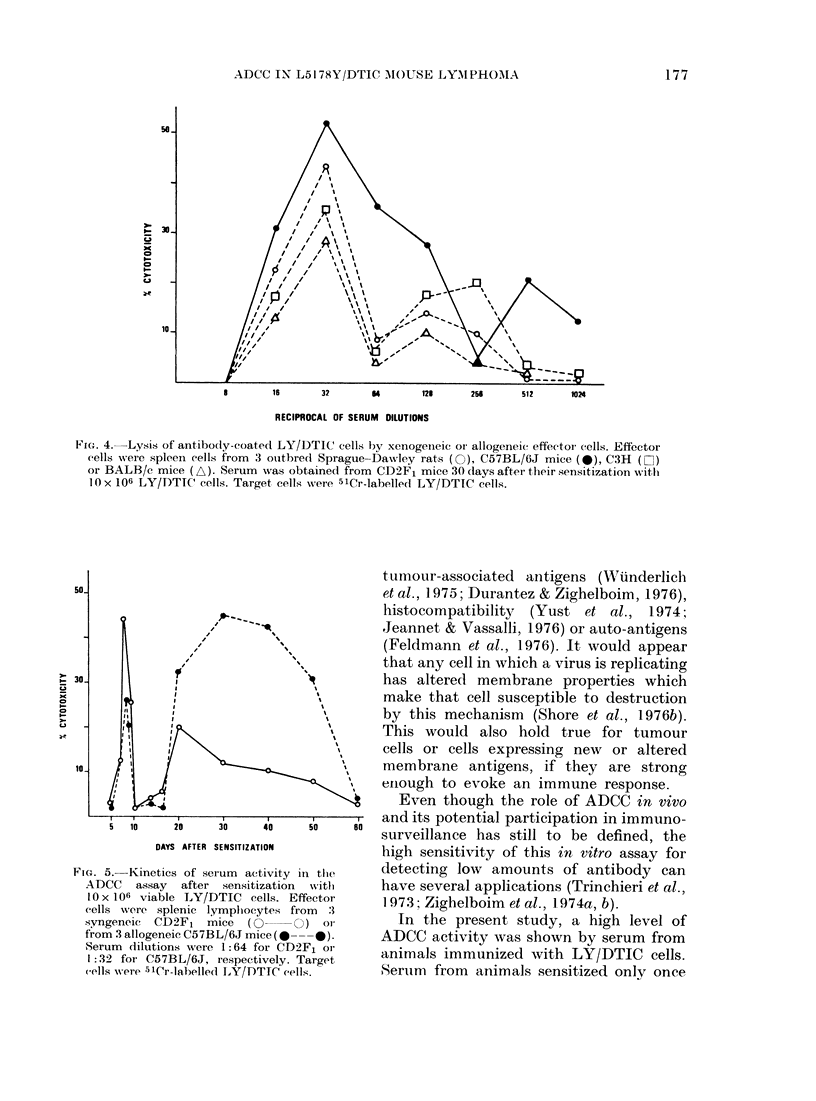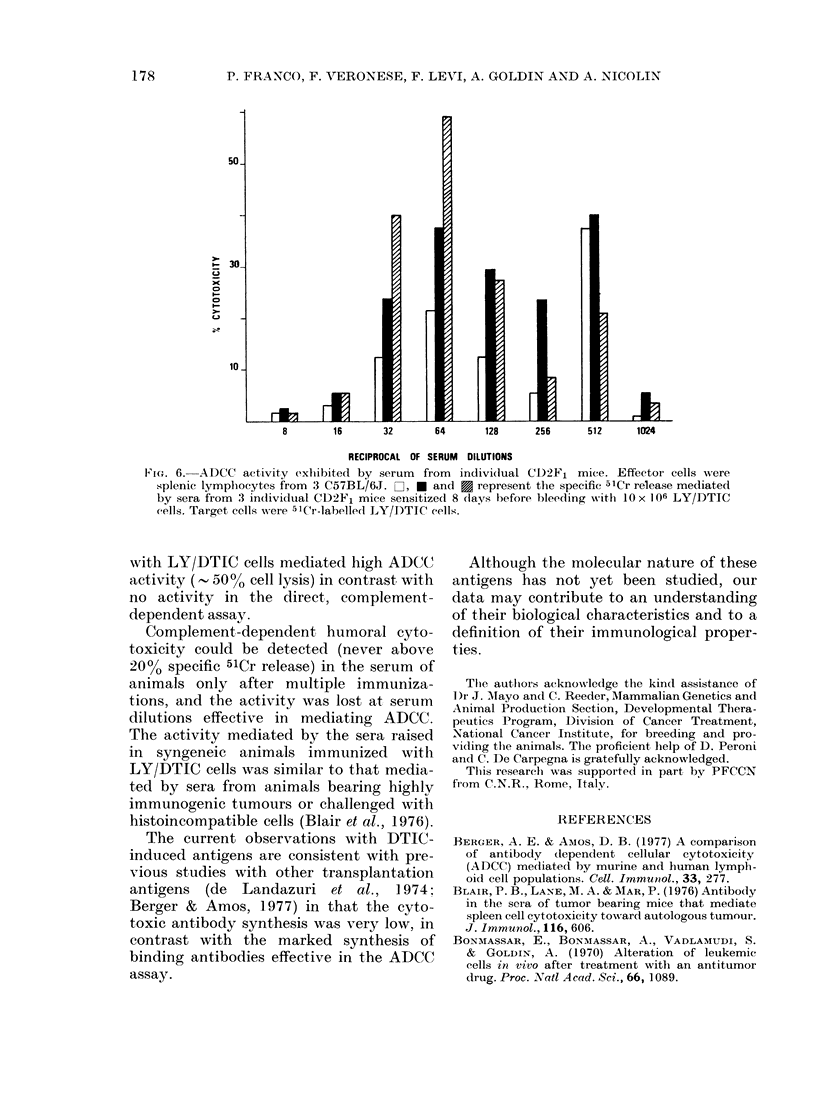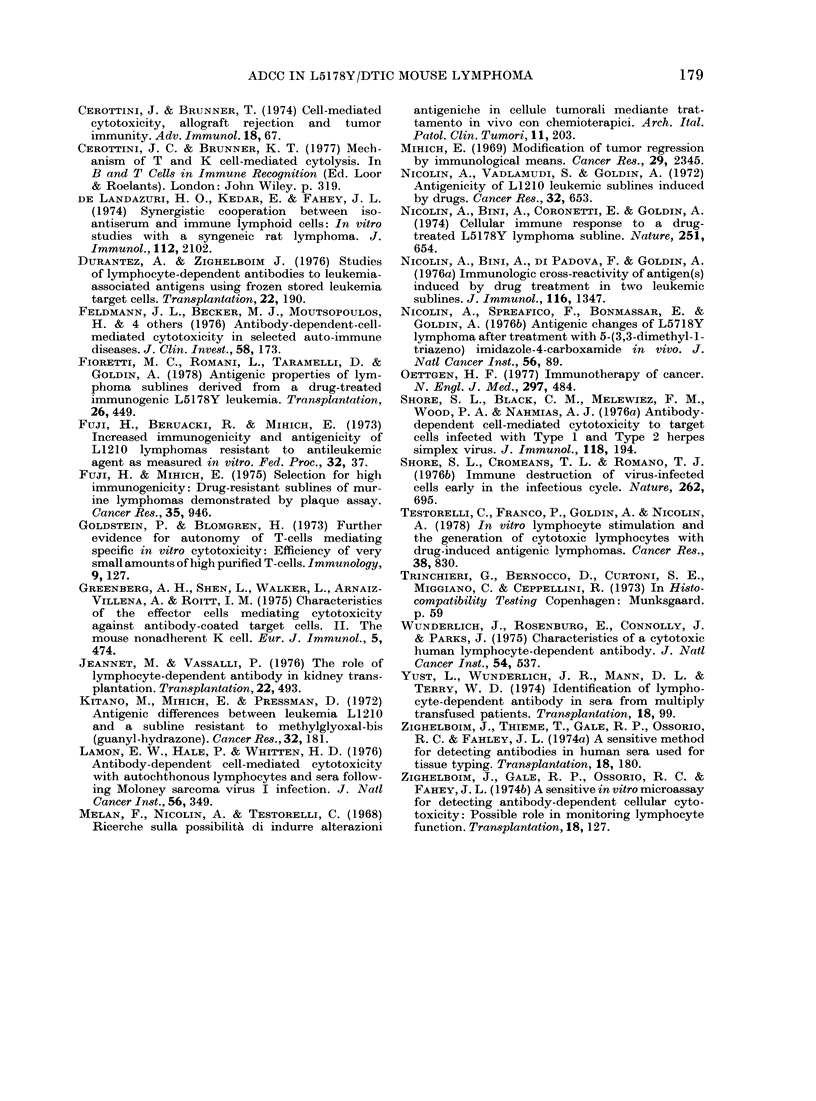# Antibody-dependent cellular cytotoxicity against drug-induced antigens in L5178Y mouse lymphoma.

**DOI:** 10.1038/bjc.1982.181

**Published:** 1982-08

**Authors:** P. Franco, F. Veronese, F. Levi, A. Goldin, A. Nicolin

## Abstract

In vivo treatment with antineoplastic compounds has been reported to lead to the expression of new antigenic specificities which were not detected on parental cells, and which were transmissible as a genetic character. The current study is concerned with antibody-dependent cellular cytotoxic (ADCC) activity in serum of syngeneic mice challenged with LY/DTIC cells, a subline of LY murine lymphoma, antigenically altered by the drug DTIC. LY/DTIC target cells coated with LY/DTIC-immune serum were specifically lysed by virgin lymphocytes. The genetic background of the effector cells, whether syngeneic, allogeneic or xenogeneic, did not produce significant differences in the percentage of target-cell lysis. ADCC activity was reduced when the immune serum was added directly to the incubation medium, without precoating. Although sera from individual animals exhibited different levels of ADCC activity, they nevertheless followed the general trend of the pooled sera. Peak activity of ADCC was obtained in the sera collected on Days 8 and 30 after LY/DTIC cell challenge. The ADCC activity elicited by LY/DTIC cells may contribute to the rejection of drug-altered tumour cells.


Br. J. Cancer (1982) 46, 173

### ANTIBODY-DEPENDENT CELLULAR CYTOTOXICITY AGAINST DRUG-INDUCED ANTIGENS IN L5178Y MOUSE LYMPHOMA

P. FRANCO*, F. VERONESE*, F. LEVI*, A. GOLDIN† AND A. NICOLIN‡

*From the *Institute of Pharmacology, School of Medicine, Via Vanvitelli 32, Milan, Italy, the †N.C.I., N.I.H., Bethesda, Md, U.S.A. and ‡Institute for Cancer Research, Genoa, Italy*

Received 23 December 1981 Accepted 7 April 1982

**Summary.—In vivo treatment with antineoplastic compounds has been reported to lead to the expression of new antigenic specificities which were not detected on parental cells, and which were transmissible as a genetic character. The current study is concerned with antibody-dependent cellular cytotoxic (ADCC) activity in serum of syngeneic mice challenged with LY/DTIC cells, a subline of LY murine lymphoma, antigenically altered by the drug DTIC. LY/DTIC target cells coated with LY/DTIC- immune serum were specifically lysed by virgin lymphocytes. The genetic background of the effector cells, whether syngeneic, allogeneic or xenogeneic, did not produce significant differences in the percentage of target-cell lysis. ADCC activity was reduced when the immune serum was added directly to the incubation medium, without precoating. Although sera from individual animals exhibited different levels of ADCC activity, they nevertheless followed the general trend of the pooled sera. Peak activity of ADCC was obtained in the sera collected on Days 8 and 30 after LY/DTIC cell challenge. The ADCC activity elicited by LY/DTIC cells may contribute to the rejection of drug-altered tumour cells.**

*IN VIVO* TREATMENT with antineoplastic drugs has been found to increase the antigenicity of mouse tumour cells (Melan *et al.*, 1968; Mihich, 1969; Bonmassar *et al.*, 1970; Nicolin *et al.*, 1972, Fuji *et al.*, 1973). These new antigenic specificities were not detected on parental cells, and were transmissible, as a genetic character, after the withdrawal of the drug (Nicolin *et al.*, 1974). The pharmacologically induced antigenic alteration of tumour cells may lead to their rejection on inoculation into syngeneic hosts. An understanding of the immune reactivity to drug-induced antigens might lead to new methodologies for the experimental immunotherapy of murine neoplasms, designed to eradicate cancer cells totally (Oettgen, 1977). In a series of previous studies, drug-xenogenized tumour cells were found to elicite cellular rather than humoral immune responses (Testorelli *et al.*, 1978; Fioretti *et al.*, 1978). Antibodies specific to drug-induced antigens have been detected by a number of assays, including binding of radioiodinated immunoglobulin of hyperimmunized syngeneic animals (Kitano *et al.*, 1972), a modified plaque assay (Fuji & Mihich, 1975) and direct, complement-dependent cytotoxicity (Nicolin *et al.*, 1976*a*). In these studies, the activity, though specific and reproducible, was generally not extensive. In the present study, the serum of mice challenged with L5178Y lymphoma cells antigenically altered by treatment with the anticancer drug 5(3,3-dimethyl triazeno) imidazole-4-carboxamide (DTIC) was used to determine the extent of antibody-dependent cellular cytotoxicity (ADCC) against the DTIC-altered tumour cells.

### MATERIALS AND METHODS

*Animals and tumours.*—Three-month-old inbred DBA/2Cr, BALB/c, C57BL/6J, C3H male mice, hybrid (BALB/c × DBA/2Cr) $F_1$ male mice, hereafter called $CD2F_1$, obtained from the Charles River Breeding Laboratories, Calco, Italy, and 12–15 week-old Sprague–Dawley male rats obtained from Farmitalia-Carlo Erba, Milan, Italy, were used in the experiments. L5178Y lymphoma (LY) chemically induced in DBA/2 mice (kindly supplied by Dr P. Alexander, Sutton, England) was maintained by weekly i.p. passages in compatible animals. The LY/DTIC subline was obtained by daily *in vivo* treatment of the parental LY lymphoma with DTIC (100 mg/kg i.p.) for 5 transplant generations, as previously described (Nicolin *et al.*, 1976*b*), and maintained by *in vivo* passages in $CD2F_1$ mice that had been immunosuppressed with cyclophosphamide (200 mg/kg i.p.) 24 h before tumour inoculation.

*Drugs.*—DTIC (NSC-45388) was dissolved in chilled saline after addition of equal parts (w/w) of citric acid. Cyclophosphamide (NSC-26271) was dissolved in saline immediately before use. Both drugs were obtained through the courtesy of Dr V. L. Narayanan, Developmental Therapeutics Program, Division of Cancer Treatment, National Cancer Institute, Bethesda, Maryland, U.S.A.

*Immune sera.*—$CD2F_1$ mice were immunized against LY/DTIC tumour cells by i.p. challenge with $10^7$ viable cells or to LY tumour cells by i.p. challenge of $2 \times 10^7$ irradiated cells (Securix Compact machine; 50 Gy 200 kV, 12 mA, 0·5 mm Cu-Al filter 1 Gy/min). Blood was obtained from the retro-orbital sinus at the time indicated, and sera were heat-inactivated at 56 °C for 45 min before use. Hyperimmune sera were obtained as above, but using 6 immunizations at 2-week intervals. In this case the first sensitization was s.c. with a 50:50 mixture (v/v) of cell suspension and complete Freund adjuvant.

*ADCC assay.*—The $^{51}Cr$-release microassay (Goldstein & Blomgren, 1973) was used. Target cells were LY or LY/DTIC, prelabelled with $Na_2\,^{51}CrO_4$ (Amersham) 200 $\mu$Ci/$10^7$ cells, and effector cells were spleen cells from virgin animals or circulating lymphocytes from healthy donors. $^{51}Cr$-labelled target cells ($10^5$) in a volume of 100 $\mu$l, pre-incubated with serial dilutions of the serum, were seeded in V-bottomed microplates (Sterilin, Teddington, Middlesex) with 100 $\mu$l of different ratios of effector cells and incubated 4 h at 37 °C in a moist atmosphere of 95% air and 5% $CO_2$. Alternatively, target cells and effector cells, in 100 $\mu$l final volume, were incubated 4 h in the presence of 100 $\mu$l of serum. The plates were centrifuged and 100 $\mu$l supernatant was counted in an automatic gamma counter.

The ADCC activity was expressed as percentage of cytotoxicity as follows:

$$\text{Specific cytotoxicity} = \frac{\text{ct/min in experimental samples} - \text{ct/min in controls*}}{\text{ct/min in detergent samples} - \text{ct/min in controls}} \times 100$$

### RESULTS

Serum of animals challenged with a single inoculum of $10 \times 10^6$ viable LY/DTIC cells did not exhibit complement-dependent cytotoxicity, but lytic antibodies were obtained after extensive sensitization with LY/DTIC tumour cells. In that case, however, specific $^{51}Cr$ release did not exceed that of the controls by more than 20%, and diminished rapidly on dilution (Fig. 1). Parental LY target cells were not lysed by anti-LY/DTIC serum and guinea-pig complement, nor were either cell types lysed by sera obtained in syngeneic animals by several injections of X-inactivated LY cells.

LY/DTIC target cells, coated with serum from animals challenged with $10 \times 10^6$ viable LY/DTIC cells, were lysed by virgin, syngeneic lymphocytes (Fig. 2). High and biphasic ADCC activity was obtained. A peak of target-cell lysis (about 50%) was detected at a serum dilution of 1/64, and a second peak was seen at a dilution of 1/512. A "prozone" effect, reduced ADCC activity at the

*Controls were labelled target cells incubated with normal mouse serum and spleen cells from virgin animals. Detergent for the maximum-release determination was a 5% water solution of Brij 35 (BDH, Milan, Italy).

FIG. 1.—Complement-dependent lytic activity of anti-LY/DTIC serum. $^{51}Cr$-labelled LY/DTIC cells (○), or $^{51}Cr$-labelled LY cells (●), were incubated with immune serum to LY/DTIC cells (- - -), hyper-immune serum to LYDTIC (——) and normal serum (—.—.) in the presence of complement. X-ray-inactivated parental LY cells injected in $CD2F_1$ mice did not elicit serum effective in the complement-dependent lysis, 1:5 rabbit serum pre-adsorbed on mouse liver cells was the source of complement.

highest serum concentrations, as reported by other authors in different tumour systems, and a fall of activity at serum dilutions between 1/64 and 1/512 were seen. Lysis did not occur with LY target cells or unrelated target cells, nor were X-ray-inactivated LY cells able to elicit immune sera with ADCC activity (data reported here).

Fig. 3 show that cell lysis was obtained when the ADCC assay was conducted in the presence of serum during the 4h inoculation. Although the pattern of lytic activity was similar to that obtained with precoating target cells before the incubation with effector cells (Fig. 2) the specific percentage of $^{51}Cr$ release was significantly reduced.

The genetic background of the effector cells did not substantially alter the results of the ADCC assay. As shown in Fig. 4, the pattern of the reaction was maintained, though the peak target-cell lysis was obtained at a lower dilution (1/32). The specificity was mediated by anti-LY/DTIC antibodies, while unprimed cells from different species have been the $Fc^+$ populations, antibody-dependent for cell lysis. Anti-LY serum did not show ADCC activity in the assay with allogeneic or xenogeneic lymphocytes (data not reported here). The kinetics of antibody production after challenge with LY/DTIC cells is reported in Fig. 5. With syngeneic or allogeneic effector cells, two peaks of activity were observed: a narrow peak on Day 8 after the immunizing inoculum, and a second, broad peak around the 30th day. Maximum activity was obtained on Day 8 with syngeneic effectors, and on Day 30 with allogeneic lymphocytes. Comparison of the differences in activity is not possible, since target cells were coated with serum diluted 1/64 or 1/32, depending on whether the effector system was syngeneic or allogeneic. Fig. 6 shows the ADCC activity of serum from individual animals. Target cells coated with serum from $CD2F_1$ mice challenged with $10 \times 10^6$ LY/DTIC cells i.p. showed differences in susceptibility to lysis by C57BL/6J lymphocytes. Despite the individual variability of the sera in mediating *in vitro* ADCC, the pattern of the activity was similar to that observed with pooled sera.

### DISCUSSION

It is well established that target-cell destruction can be mediated through different immunological mechanisms (Cerottini & Brunner, 1977). Specific cellular lysis of tumour cell is mainly performed by the classic T-cell immunity mechanism (Cerottini & Brunner, 1974) or the interaction of antibody with normal lymphoid cells, the latter being referred to as antibody-dependent cellular cytotoxicity (ADCC). The precise mechanism of ADCC is not understood, though it is known that the specificity of this imimunological reaction is determined by antibody bound to membrane-associated

FIG. 2.—Specific ADCC activity of anti-LY/DTIC serum. Effector cells were splenic lymphocytes from $CD2F_1$ mice. Sera, obtained by bleeding 3 $CD2F_1$ mice 8 days after i.p. challenge with $10 \times 10^6$ LY/DTIC cells or with $10 \times 10^6$ X-ray-inactivated LY cells (40 Gy) were heat-inactivated before use. ADCC anti-LY/DTIC serum was checked against LY/DTIC cells (●—●), LY cells (○- - -○), L1210 cells (△- - -△) and EL4 cells (□- - -□). ADCC anti-LY serum was checked against LY/DTIC cells (●- - -●) and LY cells (○- - -○). The prebleeds tested against target cells were never above the background.

FIG. 3.—Reduced ADCC activity on adding the antiserum to effector and target cells during the 4h pre-incubation. Effector cells were spleen cells from $CD2F_1$ mice. Serum was obtained as indicated in Fig. 2. $^{51}Cr$-labelled targets were LY/DTIC cells (○) or LY cells (●).

antigens, and that the effector cells bear receptors for the $F_c$ fragment of the immunoglobulin molecules (Greenberg *et al.*, 1975), but the effector cells lack both T- and B-cell characteristics.

Little is known about the *in vivo* significance of ADCC, which was identified by means of *in vitro* experiments, both in human and animal systems. ADCC has been demonstrated against cells infected with a variety of viruses (Lamon *et al.*, 1976; Shore *et al.*, 1976*a*) and also against

FIG. 4.—Lysis of antibody-coated LY/DTIC cells by xenogeneic or allogeneic effector cells. Effector cells were spleen cells from 3 outbred Sprague–Dawley rats (○), C57BL/6J mice (●), C3H (□) or BALB/c mice (△). Serum was obtained from $CD2F_1$ mice 30 days after their sensitization with $10 \times 10^6$ LY/DTIC cells. Target cells were $^{51}Cr$-labelled LY/DTIC cells.

FIG. 5.—Kinetics of serum activity in the ADCC assay after sensitization with $10 \times 10^6$ viable LY/DTIC cells. Effector cells were splenic lymphocytes from 3 syngeneic $CD2F_1$ mice (○——○) or from 3 allogeneic C57BL/6J mice (●– – –●). Serum dilutions were 1:64 for $CD2F_1$ or 1:32 for C57BL/6J, respectively. Target cells were $^{51}Cr$-labelled LY/DTIC cells.

tumour-associated antigens (Wünderlich *et al.*, 1975; Durantez & Zighelboim, 1976), histocompatibility (Yust *et al.*, 1974; Jeannet & Vassalli, 1976) or auto-antigens (Feldmann *et al.*, 1976). It would appear that any cell in which a virus is replicating has altered membrane properties which make that cell susceptible to destruction by this mechanism (Shore *et al.*, 1976*b*). This would also hold true for tumour cells or cells expressing new or altered membrane antigens, if they are strong enough to evoke an immune response.

Even though the role of ADCC *in vivo* and its potential participation in immunosurveillance has still to be defined, the high sensitivity of this *in vitro* assay for detecting low amounts of antibody can have several applications (Trinchieri *et al.*, 1973; Zighelboim *et al.*, 1974*a*, *b*).

In the present study, a high level of ADCC activity was shown by serum from animals immunized with LY/DTIC cells. Serum from animals sensitized only once

FIG. 6.—ADCC activity exhibited by serum from individual CD2F$_1$ mice. Effector cells were splenic lymphocytes from 3 C57BL/6J. □, ■ and ▨ represent the specific $^{51}$Cr release mediated by sera from 3 individual CD2F$_1$ mice sensitized 8 days before bleeding with $10 \times 10^6$ LY/DTIC cells. Target cells were $^{51}$Cr-labelled LY/DTIC cells.

with LY/DTIC cells mediated high ADCC activity ($\sim 50\%$ cell lysis) in contrast with no activity in the direct, complement-dependent assay.

Complement-dependent humoral cytotoxicity could be detected (never above 20% specific $^{51}$Cr release) in the serum of animals only after multiple immunizations, and the activity was lost at serum dilutions effective in mediating ADCC. The activity mediated by the sera raised in syngeneic animals immunized with LY/DTIC cells was similar to that mediated by sera from animals bearing highly immunogenic tumours or challenged with histoincompatible cells (Blair *et al.*, 1976).

The current observations with DTIC-induced antigens are consistent with previous studies with other transplantation antigens (de Landazuri *et al.*, 1974; Berger & Amos, 1977) in that the cytotoxic antibody synthesis was very low, in contrast with the marked synthesis of binding antibodies effective in the ADCC assay.

Although the molecular nature of these antigens has not yet been studied, our data may contribute to an understanding of their biological characteristics and to a definition of their immunological properties.

The authors acknowledge the kind assistance of Dr J. Mayo and C. Reeder, Mammalian Genetics and Animal Production Section, Developmental Therapeutics Program, Division of Cancer Treatment, National Cancer Institute, for breeding and providing the animals. The proficient help of D. Peroni and C. De Carpegna is gratefully acknowledged.

This research was supported in part by PFCCN from C.N.R., Rome, Italy.

### REFERENCES

BERGER, A. E. & AMOS, D. B. (1977) A comparison of antibody dependent cellular cytotoxicity (ADCC) mediated by murine and human lymphoid cell populations. *Cell. Immunol.*, **33**, 277.

BLAIR, P. B., LANE, M. A. & MAR, P. (1976) Antibody in the sera of tumor bearing mice that mediate spleen cell cytotoxicity toward autologous tumour. *J. Immunol.*, **116**, 606.

BONMASSAR, E., BONMASSAR, A., VADLAMUDI, S. & GOLDIN, A. (1970) Alteration of leukemic cells *in vivo* after treatment with an antitumor drug. *Proc. Natl Acad. Sci.*, **66**, 1089.

CEROTTINI, J. & BRUNNER, T. (1974) Cell-mediated cytotoxicity, allograft rejection and tumor immunity. *Adv. Immunol.* **18**, 67.

CEROTTINI, J. C. & BRUNNER, K. T. (1977) Mechanism of T and K cell-mediated cytolysis. In *B and T Cells in Immune Recognition* (Ed. Loor & Roelants). London: John Wiley. p. 319.

DE LANDAZURI, H. O., KEDAR, E. & FAHEY, J. L. (1974) Synergistic cooperation between isoantiserum and immune lymphoid cells: *In vitro* studies with a syngeneic rat lymphoma. *J. Immunol.*, **112**, 2102.

DURANTEZ, A. & ZIGHELBOIM J. (1976) Studies of lymphocyte-dependent antibodies to leukemia-associated antigens using frozen stored leukemia target cells. *Transplantation*, **22**, 190.

FELDMANN, J. L., BECKER, M. J., MOUTSOPOULOS, H. & 4 others (1976) Antibody-dependent-cell-mediated cytotoxicity in selected auto-immune diseases. *J. Clin. Invest.*, **58**, 173.

FIORETTI, M. C., ROMANI, L., TARAMELLI, D. & GOLDIN, A. (1978) Antigenic properties of lymphoma sublines derived from a drug-treated immunogenic L5178Y leukemia. *Transplantation*, **26**, 449.

FUJI, H., BERUACKI, R. & MIHICH, E. (1973) Increased immunogenicity and antigenicity of L1210 lymphomas resistant to antileukemic agent as measured *in vitro*. *Fed. Proc.*, **32**, 37.

FUJI, H. & MIHICH, E. (1975) Selection for high immunogenicity: Drug-resistant sublines of murine lymphomas demonstrated by plaque assay. *Cancer Res.*, **35**, 946.

GOLDSTEIN, P. & BLOMGREN, H. (1973) Further evidence for autonomy of T-cells mediating specific *in vitro* cytotoxicity: Efficiency of very small amounts of high purified T-cells. *Immunology*, **9**, 127.

GREENBERG, A. H., SHEN, L., WALKER, L., ARNAIZ-VILLENA, A. & ROITT, I. M. (1975) Characteristics of the effector cells mediating cytotoxicity against antibody-coated target cells. II. The mouse nonadherent K cell. *Eur. J. Immunol.*, **5**, 474.

JEANNET, M. & VASSALLI, P. (1976) The role of lymphocyte-dependent antibody in kidney transplantation. *Transplantation*, **22**, 493.

KITANO, M., MIHICH, E. & PRESSMAN, D. (1972) Antigenic differences between leukemia L1210 and a subline resistant to methylglyoxal-bis (guanyl-hydrazone). *Cancer Res.*, **32**, 181.

LAMON, E. W., HALE, P. & WHITTEN, H. D. (1976) Antibody-dependent cell-mediated cytotoxicity with autochthonous lymphocytes and sera following Moloney sarcoma virus I infection. *J. Natl Cancer Inst.*, **56**, 349.

MELAN, F., NICOLIN, A. & TESTORELLI, C. (1968) Ricerche sulla possibilità di indurre alterazioni antigeniche in cellule tumorali mediante trattamento in vivo con chemioterapici. *Arch. Ital. Patol. Clin. Tumori*, **11**, 203.

MIHICH, E. (1969) Modification of tumor regression by immunological means. *Cancer Res.*, **29**, 2345.

NICOLIN, A., VADLAMUDI, S. & GOLDIN, A. (1972) Antigenicity of L1210 leukemic sublines induced by drugs. *Cancer Res.*, **32**, 653.

NICOLIN, A., BINI, A., CORONETTI, E. & GOLDIN, A. (1974) Cellular immune response to a drug-treated L5178Y lymphoma subline. *Nature*, **251**, 654.

NICOLIN, A., BINI, A., DI PADOVA, F. & GOLDIN, A. (1976*a*) Immunologic cross-reactivity of antigen(s) induced by drug treatment in two leukemic sublines. *J. Immunol.*, **116**, 1347.

NICOLIN, A., SPREAFICO, F., BONMASSAR, E. & GOLDIN, A. (1976*b*) Antigenic changes of L5718Y lymphoma after treatment with 5-(3,3-dimethyl-1-triazeno) imidazole-4-carboxamide *in vivo*. *J. Natl Cancer Inst.*, **56**, 89.

OETTGEN, H. F. (1977) Immunotherapy of cancer. *N. Engl. J. Med.*, **297**, 484.

SHORE, S. L., BLACK, C. M., MELEWIEZ, F. M., WOOD, P. A. & NAHMIAS, A. J. (1976*a*) Antibody-dependent cell-mediated cytotoxicity to target cells infected with Type 1 and Type 2 herpes simplex virus. *J. Immunol.*, **118**, 194.

SHORE, S. L., CROMEANS, T. L. & ROMANO, T. J. (1976*b*) Immune destruction of virus-infected cells early in the infectious cycle. *Nature*, **262**, 695.

TESTORELLI, C., FRANCO, P., GOLDIN, A. & NICOLIN, A. (1978) *In vitro* lymphocyte stimulation and the generation of cytotoxic lymphocytes with drug-induced antigenic lymphomas. *Cancer Res.*, **38**, 830.

TRINCHIERI, G., BERNOCCO, D., CURTONI, S. E., MIGGIANO, C. & CEPPELLINI, R. (1973) In *Histocompatibility Testing* Copenhagen: Munksgaard. p. 59

WUNDERLICH, J., ROSENBURG, E., CONNOLLY, J. & PARKS, J. (1975) Characteristics of a cytotoxic human lymphocyte-dependent antibody. *J. Natl Cancer Inst.*, **54**, 537.

YUST, L., WUNDERLICH, J. R., MANN, D. L. & TERRY, W. D. (1974) Identification of lymphocyte-dependent antibody in sera from multiply transfused patients. *Transplantation*, **18**, 99.

ZIGHELBOIM, J., THIEME, T., GALE, R. P., OSSORIO, R. C. & FAHLEY, J. L. (1974*a*) A sensitive method for detecting antibodies in human sera used for tissue typing. *Transplantation*, **18**, 180.

ZIGHELBOIM, J., GALE, R. P., OSSORIO, R. C. & FAHEY, J. L. (1974*b*) A sensitive *in vitro* microassay for detecting antibody-dependent cellular cytotoxicity: Possible role in monitoring lymphocyte function. *Transplantation*, **18**, 127.